# Association between peer relationships and exercise self-efficacy among college students: the mediating role of physical activity commitment

**DOI:** 10.3389/fpsyg.2025.1533097

**Published:** 2025-08-05

**Authors:** Han Bao

**Affiliations:** Department of Physical Education, Nanjing University of Industry Technology, Nanjing, Jiangsu, China

**Keywords:** college student population, peer relationships, exercise self-efficacy, physical activity engagement, mediating role

## Abstract

**Purpose:**

This study investigates the correlation between peer relationships and exercise self-efficacy among college students and examines the mediating role of physical activity inputs. These findings aim to enhance college students’ physical activity levels and promote their overall physical and mental wellbeing.

**Methods:**

The Peer Relationship Scale, Exercise Self-Efficacy Scale, and Physical Activity Behavioral Intention Scale were included in a questionnaire administered to 514 university students on 12 July 2024.

**Results:**

(1) There were no sex differences in peer relationships among college students (*p* > 0.05), but there was a sex difference in exercise self-efficacy and physical activity commitment (*p* < 0.05), with male students demonstrating higher levels than female students. (2) Peer relationships were positively correlated with exercise self-efficacy (*r* = 0.832, *p* < 0.05); peer relationships were positively correlated with physical activity commitment (*r* = 0.743, *p* < 0.05); and exercise self-efficacy was positively correlated with physical activity commitment (*r* = 0.758, *p* < 0.05). (3) The direct effect of college students’ peer relationships on exercise self-efficacy was significant, with an effect size of 74.26%. (4) The mediating effect of physical activity commitment between college students’ peer relationships and exercise self-efficacy was significant, with an effect size of 25.74%.

**Conclusion:**

(1) There is a close relationship between college students’ peer relationships, exercise self-efficacy, and physical activity inputs. (2) Peer relationships significantly influence college students’ exercise self-efficacy, and they can also have an indirect effect on exercise self-efficacy through the pathway of action of physical activity input.

## 1 Introduction

In recent years, the Chinese Government has attached great importance to the physical health of students. The 20th National Congress of the Communist Party of China clearly states that “Efforts in school health and health education should focus on holistically enhancing students’ health. Additionally, it is imperative to strengthen the school health system and bolster capacity-building initiatives” (Education, 2021). Physical exercise refers to physical activities in which individuals actively participate to improve their health, motor skills, and good exercise behavior (Barnard, 2009). Moreover, it serves as a crucial means to improve college students’ physical and mental wellbeing (Zhang et al., 2021). However, the physical inactivity of adolescents/young people is a major global challenge, with World Health Organization (WHO) data showing that more than 80 per cent of adolescents fail to meet the recommended daily activity levels, a trend that is particularly pronounced among university students (WHO, 2019). In China, survey data from the Youth Net Campus News Agency further confirm that the proportion of university students exercising less than three times a week is as high as 48.19 per cent, with 58.7 per cent exercising for no more than 30 min at a time (Daily, 2020, 2023).

College students’ commitment to physical activity is influenced by the confluence of these factors. To illustrate, academic pressure, interpersonal environment (e.g., peer relationships), and individual perceptions (e.g., exercise self-efficacy) have been widely recognized as important predictors (El Masri et al., 2021; Salvesen et al., 2008; Shum et al., 2022). Recent research by domestic and international scholars has revealed a correlation between peer relationships, exercise self-efficacy, and physical activity (Chen et al., 2017; Kooijmans et al., 2020). An experimental study by the International Association for Physical Education (IAPE) found that good peer relationships significantly increased college students’ exercise self-efficacy, which in turn increased their commitment to physical activity (Zou et al., 2023). So, how do the interaction mechanisms between peer relationships, physical activity engagement, and exercise self-efficacy manifest in college student populations? Can peer relationships promote physical activity engagement and enhance individual exercise self-efficacy? Clarifying these questions is conducive to exploring the relationship between many factors influencing college students’ physical activity, which is an important part of promoting the development of college students’ physical and mental health and an important issue that needs to be addressed by academics. Therefore, this study hypothesizes that there is a correlation between peer relationships, exercise self-efficacy, and physical activity commitment and makes the following statements about the specific hypotheses.

### 1.1 Relationship between peer relationships and exercise self-efficacy

The term “peer relationship” is used to describe the interpersonal dynamics that emerge when individuals of a similar age or level of psychological development engage in shared activities or collaborative endeavors (Mounts, 2011). This is an important factor influencing socialization in the process of individual growth (Zhou et al., 2023). Good peer relationships can provide college students with the necessary emotional support (Sjolie et al., 2024), reduce stress from study and work, and lower the probability of depression (Li et al., 2020). Furthermore, it has been demonstrated to enhance exercise self-efficacy. From the perspective of self-efficacy theory, individuals’ self-efficacy, or belief in their ability to succeed, affects the degree of expectation of success that they hold (Calaguas and Consunji, 2022). Among college students, a higher level of self-efficacy is associated with a greater aspiration for success. Similarly, in the process of physical exercise, exercise self-efficacy also affects an individual’s desire to achieve physical exercise goals. A positive correlation exists between exercise self-efficacy and the strength of the desire to achieve exercise goals among college students. Previous research has shown that exercise self-efficacy is influenced by multiple factors such as exercise experience, skill level, physical intention, social support, and peer relationships (Almutary and Tayyib, 2020; Bani-Issa et al., 2020; O'Neil-Pirozzi, 2021). Additionally, studies have indicated a correlation between peer relationships and exercise self-efficacy (Chen and Zheng, 2021), as demonstrated by the fact that peer friendship positively predicts exercise self-efficacy (Zhang et al., 2022). Based on this finding, the present study proposed Hypothesis H1: Peer relationships can positively predict exercise self-efficacy.

### 1.2 Mediating effect of physical activity engagement between peer relationships and exercise self-efficacy

Physical activity commitment refers to the amount of time, energy, and effort that an individual puts into physical activity (Hagberg et al., 2020). A number of studies have demonstrated that an individual’s level of physical activity commitment is influenced by multiple factors, including their personal interests, the social environment in which they live, and the exercise program they adhere to (El Masri et al., 2021; Salvesen et al., 2008; Yang et al., 2021). Self-determination theory suggests that the social environment can enhance individual motivation and its associated transformations through the fulfillment of the three basic psychological needs of supportive relationships, autonomy, and competence (Hui and Tsang, 2012). Some studies have further pointed out that peer relationships have a positive predictive effect on physical activity engagement. It can be reasonably deduced that, as the level of peer relationships increases, the level of physical activity engagement among college students also increases (Li et al., 2016). Through physical activity, college students can obtain certain support and encouragement and learn various emotional competencies in the process of communicating with their peers, thereby enhancing their engagement in physical activity (Bi et al., 2023). At the same time, communication between peers during the exercise process also helps establish and strengthen social connections, promote peer relationships, and enhance the enjoyment of physical exercise (Lee et al., 2022). Another empirical study also proves the above point that good peer relationships can promote college students’ engagement in physical activity and make them more active in physical activity; thus, peer emotional support (e.g., peer praise and companionship) can help to enhance individual motivation for physical activity and maintain a certain level of intensity of physical activity behavior (Pierannunzio et al., 2022). This indicates that college students need to establish good peer relationships to increase their commitment to physical activity and maintain a healthy lifestyle through mutual encouragement and support from their peers. On this basis, this study proposes Hypothesis H2a: Peer relationships have a significant positive predictive effect on college students’ physical activity engagement.

**Figure 1:**
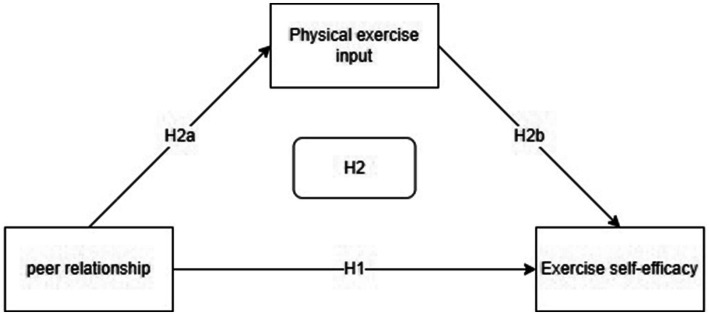
Conceptual architecture model.

Self-efficacy can be defined as an individual’s subjective judgment, confidence, and beliefs regarding their capacity to successfully attain a behavioral objective (Calaguas and Consunji, 2022). In a constrained exercise context, exercise self-efficacy can be defined as an individual’s cognitive capacity to believe that they will be able to achieve objectives and tasks associated with physical activity. It can be posited that individuals with higher exercise self-efficacy are better equipped to regulate and manage their own behavior (Neace et al., 2022). Some studies have pointed out that there is an interactive relationship between physical activity input and exercise self-efficacy (Hu et al., 2016). On the one hand, the positive physical activity input of individuals can enrich the experience and accomplishment of exercise, which is manifested in the fact that college students can gradually enhance their athletic ability by participating in physical activity, thus improving their sense of athletic self-efficacy (Chu et al., 2021). On the other hand, from the standpoint of self-efficacy, believing in one’s capability to engage in physical activity can serve as a source of emotional stability and motivation. College students who possess high levels of exercise self-efficacy tend to more motivated to engage in physical activity (Tao et al., 2024). This shows that there is a positive correlation between physical activity engagement and exercise self-efficacy in the college student population. Thus, this study proposes Hypothesis H2b: Physical activity input can significantly and positively predict exercise self-efficacy. The review indicates a strong correlation between peer relationships and exercise self-efficacy, as well as a significant relationship between physical activity input and exercise self-efficacy. Therefore, Hypothesis H2: Physical activity input plays a significant mediating role between peer relationships and exercise self-efficacy is further proposed. We assume that the model is as shown in Figure 1.

## 2 Participants

In this study, due to time and resource constraints, 512 freshmen, sophomores, and juniors from three colleges and universities in a city were selected as survey respondents through convenience sampling. This method facilitated efficient data collection. However, it poses limitations on the generalizability of the results. Future studies should incorporate stratified sampling to enhance representation.

With the help of counselors and following the principle of voluntariness, from 12 July 2024, to 14 July 2024, the group survey was conducted, and the subjects were informed about the confidentiality of their responses before taking the test and started answering only after they agreed. A total of 549 paper questionnaires were distributed, 35 regular and unanswered questionnaires were excluded, and 514 valid questionnaires were recovered (M ± SD = 19.01 ± 1.27), with a valid recovery rate of 93.6%. The effective recovery rate was 93.6%. Among them, the number of male students was 285, accounting for 55.5%, and 229 female students, accounting for 44.5% (Table 1).

**Table 1:** Distribution of demographic variables among survey respondents.

Variant	Form	Quorum	Percentage (%)
Sex	Men	285	55.4
	Female	229	44.6
Grade	Freshman	173	33.7
	Sophomore	170	33.1
	Third year	171	33.3
Age	18–19 years old	75	14.6
	19–20 years old	113	22.0
	20–21 years old	138	26.8
	21–22 years old	112	21.8
	22 years old and over	76	14.8
Total		514	100

## 3 Methods

### 3.1 Peer relationship scale

The Peer Relationship Scale, developed by Wei Yunhua, was employed in this study (Yunhua, 1998). It is applicable to the college student population, contains 20 questions, and is based on a 5-point Likert scale, with 1–5 representing completely incorrect, incorrect, uncertain, correct, and completely correct, respectively; the higher the score, the higher the subject’s perpetual companionship. The Cronbach’s *α* coefficient for this scale in the current study was 0.857.

### 3.2 Exercise self-efficacy scale

The Chinese version of the Exercise Self-Efficacy Scale (Chen et al., 2019), developed by Motl et al. and revised by Chinese scholar Chen et al., was used in this study. The scale has been localized to make it more relevant to the cultural background and exercise habits of Chinese university students, with eight questions and a 5-point Likert scale, ranging from “1” for complete disagreement to “5” for complete agreement. The higher the sum of the scores for all questions, the higher the students’ self-efficacy for exercise. The Cronbach’s *α* coefficient for this scale was 0.762 in this study.

### 3.3 Physical activity engagement scale

The level of college students’ physical activity behavior was selected from the physical activity behavior intention subscale of the Physical Activity Rating Scale compiled by Dq (1994). With a total of eight questions, the subscale questionnaire had good reliability and validity, and a 5-point Likert scale was used, with 1–5 representing very non-compliant, non-compliant, uncertain, compliant, and very compliant, respectively. The Cronbach’s *α* coefficient for the scale in this study was 0.808.

### 3.4 Statistical analysis

A common method bias test, descriptive statistics, correlation analysis, independent samples *t*-test, and ANOVA were performed sequentially using SPSS 27.0. Hayes’ process procedure was used to test for mediating effects and to construct a mediation model, where X = peer relationships, Y = exercise self-efficacy, and M = physical activity commitment.

## 4 Results

### 4.1 Common method bias test

The Harman one-factor test was used in this study to test for common method bias (Zhou, 2004). As a result, a total of nine factors with an eigenroot greater than 1 were extracted; the first factor cumulatively explained 29.91% of the total variance and was less than the 40% criterion, indicating that there was no serious common method bias in this study.

### 4.2 Descriptive statistics and correlation analysis

In this study, the focus was on examining the overall scores between the variables of the model, and the subdivided dimensions of the variables were no longer considered and the mean scores of the variables, as well as the Pearson correlation analysis, were used, and the results are shown in Table 1. At the time of the correlation analysis, it was found that self-efficacy was significantly positively correlated with physical activity behavior (*p* < 0.01), and peer relationships were significantly positively correlated with physical activity behavior (*p* < 0.01). Furthermore, self-efficacy demonstrated a significant positive correlation with peer relationships (*p* < 0.01). (Table 2).

**Table 2:** Mean, standard deviation, and correlation analysis.

	Peer relationship	Exercise self-efficacy	Physical activity inputs
Peer relationship	1		
Exercise self-efficacy	0.832**	1	
Physical activity inputs	0.743**	0.758**	1
M	3.041	3.092	3.103
SD	0.523	0.572	0.634

“*” Indicates *p* < 0.05, “**” Indicates *p* < 0.01.

### 4.3 Independent samples *t*-test and ANOVA

In this study, an independent samples *t*-test was conducted on the sex of the participants (Table 3). The results of Levine’s test of variance equivalence for sex indicated that peer relationships (*p* < 0.05) exhibited non-uniform variance; therefore, variance non-uniformity data were utilized. Conversely, exercise self-efficacy (*p* > 0.05) and physical activity input (*p* > 0.05) did not show significant differences. A chi-squared test was employed to assess the equality of means for sex, and the results indicated a statistically significant difference between exercise self-efficacy (*p* < 0.001) and physical activity input (*p* < 0.001). Consequently, there is a notable discrepancy between sex and both variables, whereas peer relationships appear to be unaffected by sex.

**Table 3:** Independent samples *t*-test for sex.

		Levine’s test of variance equivalence		Mean equivalence *t*-test	
Grouping variable	Implicit variable	*F*	*p*	*t*	p
Gender	Peer relationship	4.434	0.036*	1.629	0.104
	Exercise self-efficacy	1.307	0.254	3.998	<0.001***
	Physical activity inputs	0.433	0.511	5.166	<0.001**

“*” Indicates *p* < 0.05, “**” Indicates *p* < 0.01. “***” Indicates *p* < 0.001.

At the same time, an ANOVA was conducted to compare the academic segments of the measured variables (as shown in Table 4), and it was found that among the academic segments, there were no significant differences in peer relationships, exercise self-efficacy, and physical activity commitment (*p* > 0.05).

**Table 4:** ANOVA for grade.

Grouping variable	Implicit variable	Mean square	*F*	*p*
Grade	Peer relationship	0.266	0.990	0.372
	Exercise self-efficacy	0.329	1.004	0.367
	Physical activity inputs	0.006	0.015	0.985

### 4.4 Mediating effect of physical activity commitment in college students’ peer relationships and exercise self-efficacy

**Figure 2:**
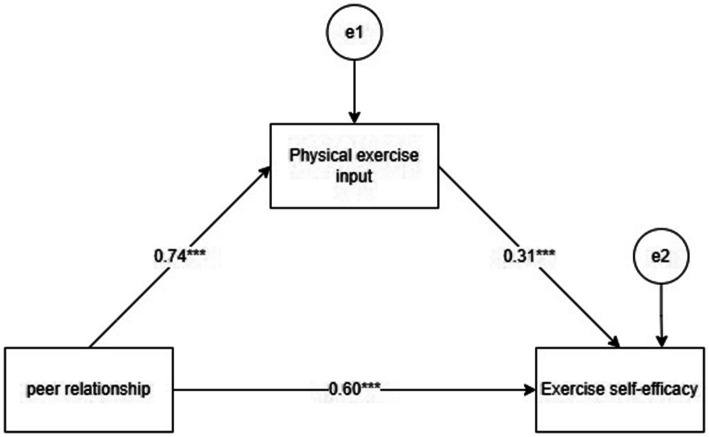
Pathways of peer relationship influence on exercise self-efficacy.

A mediation model was developed using AMOS26.0 to examine the association between physical activity input between college students’ peer relationships, exercise self-efficacy, and physical activity input (Figure 2). The analysis revealed a significant correlation between these variables, confirming that college students’ peer relationships influence exercise self-efficacy, both directly and indirectly. This relationship was further validated through the bootstrap method. Table 5 shows the mediating effects of physical activity input between peer relationship and exercise self-efficacy.

**Table 5:** Explanatory table for total, direct, and indirect effects.

	Efficiency value	SE	LLCI	ULCI	Effective quantity
Total effect	0.909	0.027	0.856	0.961	
Direct effect	0.675	0.038	0.601	0.749	74.26%
Indirect effect	0.233	0.047	0.137	0.323	25.74%

The bootstrap method was used to analyze the mediating effect of physical activity inputs on college students’ peer relationships and exercise self-efficacy, with a sample size of 5,000 selected and 95% confidence intervals. The results showed that none of the three paths had effect values with 95% CI that passed through 0, indicating that there was statistical significance, with an indirect effect value of 0.233, and the mediating effect of the bootstrap 95% confidence interval of [0.137, 0.323], which accounted for 25.74.0% of the total effect of college students’ peer relationships on exercise self-efficacy (0.909).

This suggests that more than a quarter (25.74%) of the positive effects of peer relationships on college students’ exercise self-efficacy were achieved by promoting their physical activity engagement behaviors (e.g., increasing the frequency, duration, intensity, or subjective effort level of exercise), which indicates that good peer relationships not only directly affect individuals’ exercise self-efficacy but also motivate and support their actual exercise engagement behaviors. Consequently, this sustained behavioral engagement further reinforces and enhances an individual’s confidence in their own exercise ability.

## 5 Discussion

This study reveals the direct and indirect paths of peer relationships in influencing college students’ acquisition of exercise self-efficacy, which is a positive exploration with the potential to improve their participation in physical exercise, enhance peer friendship, and promote their physical and mental wellbeing. From a theoretical perspective, this study contributes to the existing literature on the factors and mechanisms influencing college students’ exercise self-efficacy and mental health. Additionally, it advances research findings on sport psychology and school physical education. From a practical standpoint, this study illuminates the intrinsic connections among physical exercise, self-development, and peer relationships. Furthermore, it offers a novel perspective on promoting the formation of healthy exercise habits and holistic development among college students.

### 5.1 Sex differences in exercise self-efficacy and commitment to physical activity

The results of this study indicate sex differences in college students’ exercise self-efficacy and commitment to physical activity. This is because traditional Chinese society and culture often give men a stronger expectation of ‘athletic ability’ and encourage them to participate in more competitive and physically contact sports activities. This may have led to higher self-efficacy for sport among male university students, as they had accumulated more successful sport experiences during their growing-up process. Comparatively, women may have more constraints or expectations of a ‘ladylike’ image and fewer opportunities or social support to participate in certain sporting activities, which affects their sense of efficacy (DeWolfe et al., 2020). Second, there may be systematic differences between males and females in their interests and preferences for physical activity. Men may be more inclined to participate in team-based, power, or confrontational sports (e.g., basketball, football), which are typically more intense and involve longer time commitments. Women, on the other hand, may prefer individualized, non-confrontational, or flexibility-focused activities (e.g., yoga, running, dance) (Huang et al., 2022). Finally, environmental factors such as campus sports facilities, curriculum, club activities, etc., which may sometimes be unintentionally more biased toward catering to men’s sporting preferences or provide more opportunities for male-friendly activities, may also affect women’s experience of participation and engagement.

Peer relationships in this study did not show significant sex differences, suggesting that male and female undergraduates may be more similar in terms of perceived peer support, quality of friendships, or sense of social inclusion, which is in line with some research suggesting that patterns of peer relationships tend to be more diverse and individualized at the university level (Yu et al., 2023). This suggests that, despite sex differences in self-efficacy and behavioral inputs, the mechanism of action of peer relationships as an important source of social support may be of similar importance in groups of male and female students.

### 5.2 Direct effects of college students’ peer relationships on exercise self-efficacy

The results of the current study showed that college students’ peer relationships positively predicted exercise self-efficacy, with a direct effect size of 0.675, and Hypothesis H1 was valid. Research has shown that as individuals transition into adolescence, their time spent at home tends to diminish, while their reliance on peers and the value they place on peer relationships tend to increase (Shang et al., 2023; Zhou et al., 2023). According to self-efficacy theory, self-efficacy may be defined as an individual’s subjective expectations, degree of confidence, or strength of belief regarding their capacity to achieve a specific behavioral objective. This construct is shaped by a multitude of factors, including direct and alternative experiences, familial influences, educational background, and social interactions within peer groups (An et al., 2021; Jiang et al., 2020). In the college student population, the centralized nature of school teaching and learning activities allows students to interact more closely with their peers. The encouragement and support of peers play an instrumental role in influencing their belief in their capability to complete physical activity, i.e., encouragement and companionship in peer relationships promote an individual’s sense of exercise self-efficacy (Sheng et al., 2023). Prior research has further highlighted peer relationships as a significant factor in developing an individual’s self-efficacy (Ren et al., 2020). The results of the present study also build on those of previous studies by confirming that peer relationships can positively predict exercise self-efficacy, enriching previous research (Chen et al., 2017). Meanwhile, on this basis, it provides some insights into intervening in college students’ exercise self-efficacy, i.e., it can be done by enhancing college students’ peer relationships to make them believe that they can accomplish their exercise in the objectives and content.

Therefore, this study suggests that schools should select students with high exercise self-efficacy and good sports performance as peer role models to demonstrate positive exercise behaviors and skills in PE classrooms and extracurricular activities. Peer role models can inspire other students to try new sports and increase their confidence in their athletic abilities by sharing their own sports experiences and success stories. At the same time, schools can organize relevant courses or workshops to teach students how to be effective peer supporters, including how to give positive feedback, how to encourage their peers to try new sports, and how to help them overcome setbacks in sports. By developing these skills, students are better able to support their peers’ sport participation and create a positive sport atmosphere, which is important for enhancing the sport self-efficacy of the entire student body. In addition, physical education teachers and counselors were trained to increase their awareness of the role of peer relationships in physical education, as well as instructed on how to promote positive peer interactions in their daily teaching and administration. Teachers can create favorable conditions for improving students’ exercise self-efficacy by designing physical activities and curriculum content that promote peer cooperation and guide students to develop healthy peer relationships.

### 5.3 Mediating effects of physical activity inputs

Findings regarding the mediation effect indicated that, initially, peer relationships among college students serve as a positive predictor of exercise self-efficacy. This finding suggests that enhancing peer relationships within this demographic may contribute to improvements in exercise self-efficacy. Second, college students’ peer relationships can also indirectly affect exercise self-efficacy through physical activity input, as shown by the fact that college students’ peer relationships positively predicted physical activity input (H2a), and physical activity input also positively predicted exercise self-efficacy (H2b); thus, Hypothesis H2 was valid. On the one hand, the peer effect theory posits that individuals’ behaviors, emotions, and attitudes are significantly influenced by peer interactions, as people naturally observe and emulate members within their social networks. This manifests through individuals adjusting their intentions and behaviors to align with group norms and expectations (Liao et al., 2022). Empirical evidence further establishes peer relationships as a salient predictor of physical activity engagement; positive relationships enhance commitment to physical activity, whereas negative relationships diminish participation in group-based exercise (Giacobbi et al., 2014). Meanwhile, ecological model theory proposes that physical activity behaviors are influenced by multiple factors (Dunton, 2017), including individuals, families, peers, and society, and that individual physical activity commitment is also influenced by peer relationships in the social environment. Therefore, peer relationships can positively predict physical activity commitment. On the other hand, previous research has demonstrated that self-efficacy significantly influences behavior (Neace et al., 2022) and is shaped by personal experiences, environmental factors, cognitive beliefs, and goal setting. Furthermore, within the university context, individuals’ investment of resources (e.g., time and money) in physical activity may enhance their motivation to achieve exercise-related goals, thereby strengthening exercise self-efficacy (Yoon et al., 2013). Simultaneously, the engagement of college students in physical activity can enhance their experiences and accomplishments in sports. This is evidenced by the gradual improvement in athletic abilities as athletes participate in physical activities, which subsequently contributes to an increased sense of self-efficacy in the realm of sports (Kooijmans et al., 2020; Sun et al., 2025). Therefore, physical activity can positively predict exercise self-efficacy. Based on the previous discussion, this study confirmed that physical activity mediates the relationship between peer relationships and exercise self-efficacy in college students, positively influencing group exercise behavior and physical fitness development. The mediating effect of 25.74% confirms the role of physical activity engagement as a bridge between the peer environment and individual cognitive beliefs and quantitatively specifies that the positive impacts of peer relationships can be effectively transformed in real campus environments by consciously designing interventions to promote engagement in physical activity (especially within the framework of peer support), which can effectively enhance the athletic self-efficacy of college students.

In summary, this study revealed a strong association between peer relationships and exercise self-efficacy among college students and elucidated the mediating role of physical activity commitment. This finding not only enriches the theoretical framework of related fields but also provides important insights for practical applications. In the campus environment, schools can enhance exercise self-efficacy by creating a positive peer environment to motivate students to participate more in physical activities and lay a solid foundation for their physical and mental health development. For example, diversified sports activities are organized to create an encouraging sports atmosphere and provide a platform for students to showcase themselves and support each other. This will not only improve their athletic ability but also promote the formation of good exercise habits, enhance self-confidence and interpersonal skills, and ultimately achieve harmonious physical and mental development. In view of this, university administrators and educators should attach great importance to the positive role of peer relationships and develop and implement strategies to make full use of this factor to enhance students’ exercise self-efficacy and commitment to physical activity. This initiative is of inestimable value to the cultivation of well-rounded talents and the promotion of a healthy campus and is expected to open a new chapter in the study of physical education and mental health of college students, laying a solid foundation for their future.

### 5.4 Limitations of the study

Although this study revealed a close relationship between college students’ peer relationships, exercise self-efficacy, and physical activity commitment, there are some shortcomings. Future research can be conducted in the following ways.

1. The fact that this study was cross-sectional and used undergraduate students as the survey population may have limited inferences about the causal relationships among the variables. Future research could adopt a longitudinal research design to expand the sample size and examine the underlying mechanisms between the variables in the longitudinal dimension. For example, students can be followed up for several consecutive years, starting from their first year of college, to observe the dynamic process of changes in their peer relationships, exercise self-efficacy and physical activity commitment over time, as well as the interactions between these changes, thus revealing a more in-depth causal mechanism. In addition, the sample size should be enlarged to cover college students of different academic levels, grades, majors, regions, and other backgrounds as much as possible to enhance the diversity and representativeness of the sample, thereby improving the external validity of the research results, making them more widely applicable to various groups of college students, providing a more solid and reliable basis for a comprehensive and in-depth understanding of college students’ physical activity, and further promoting research and practical application in this field. This will further promote the development and practical applications of research in this field.
2. Based on the results of this study, future research can explore the importance of applying interventions to the physical and mental health development of college students. Specifically, a comprehensive intervention program can be designed and implemented from a multidimensional perspective. On the one hand, the mental health education system can be strengthened, and the psychological literacy of college students can be enhanced through systematic curricula and diversified activities. On the other hand, the structure of physical education curricula can be optimized, and innovative teaching modes and sports can be incorporated according to the needs and interests of students, while the social support network can be expanded simultaneously, and the integration of internal and external resources on and off campus can provide sufficient emotional and practical assistance to the students. The university will also expand its social support network and integrate resources inside and outside campus to provide students with sufficient emotional and practical assistance.

## 6 Conclusion

This study initially explored and clarified the complex association between peer relationships, exercise self-efficacy, and physical activity commitment and their paths of action through a survey of 514 college students. There is a significant positive association between college students’ self-efficacy, peer relationships, and physical activity behaviors. Among them, college students’ self-efficacy is an important influence on physical activity behaviors, and at the same time, it can indirectly influence college students’ physical activity behaviors through the path of peer relationships.

## Data Availability

The original contributions presented in the study are included in the article/supplementary material, further inquiries can be directed to the corresponding author.
